# The Correlation Between iNKT Cells and Malignant Tumors: A Meta-Analysis

**DOI:** 10.3390/curroncol33100579

**Published:** 2026-09-28

**Authors:** Chendi Liang, Xiaoxia Wang, Jun Lu

**Affiliations:** 1 Department of Medical Oncology, Beijing YouAn Hospital, Capital Medical University, Beijing 100069, China; 2 Laboratory for Clinical Medicine, Capital Medical University, Beijing 100069, China

**Keywords:** iNKT, cancer, prognosis, treatment, meta-analysis

## Abstract

Cancer is one of the major global health problems, with increasing numbers of new cases and deaths each year. This highlights the urgency of effective treatment. The iNKT cells act as a special subset of T cells with potent anti-tumor effects. Therefore, the correlation between iNKT cells and cancer will be evaluated in terms of frequency, prognosis, and efficacy in this study. A total of 62 studies involving 1076 healthy controls and 2453 cancer patients were included in this meta-analysis. The meta-analysis showed that the number of circulating iNKT cells was significantly higher in healthy controls than in cancer patients, as well as the frequency of iNKT cells in circulating T cells and mononuclear cells. At the same time, the frequency of iNKT cells in T cells was significantly higher in non-tumor tissues than in tumor tissues. In terms of prognosis, the higher frequency of iNKT cells in peripheral blood or tumor tissues of cancer patients was associated with improved overall survival (OS) and relapse-free survival (RFS). Regarding the efficacy of iNKT cell-related therapies, the objective response rate was 3%, and the disease control rate was 49%. In conclusion, iNKT cells were significantly reduced in cancer patients, and the higher frequency of iNKT cells was associated with a favorable prognosis in these patients.

## Simple Summary

iNKT cells are a special subset of T cells with significant anti-tumor activity, but their association with cancer remains unclear. This meta-analysis clarifies the correlation between iNKT cells and malignant tumors (including solid tumors and hematologic malignancies) from three aspects: frequency, prognosis, and therapeutic efficacy. The results showed that the frequency of iNKT cells in cancer patients was significantly reduced, a higher level of iNKT cells could be associated with improved prognosis, and iNKT cell-related therapies have demonstrated therapeutic efficacy. This provides new insights into iNKT cells and malignant tumors.

## 1. Introduction

Cancer is recognized as one of the major global health problems that seriously affects human health. In 2022, the number of new cancer cases globally was 20 million, and the number of deaths exceeded 9.7 million. Lung cancer was the most common new cancer, accounting for 12.4% of all cancers globally; the others in order were breast cancer (11.6%), colorectal cancer (9.6%), prostate cancer (7.3%), gastric cancer (4.9%), liver cancer (4.3%), and so on [1]. Additionally, hematologic tumors such as leukemia, lymphoma, and multiple myeloma are also common fatal malignant tumors, which are characterized by abnormal proliferation and the differentiation of hematopoietic cells. Due to high recurrence rates and poor prognosis, hematologic tumors still remain critical clinical challenges [2]. With the advancement of technology, the treatment mode of cancer has changed from traditional surgery, chemotherapy, and radiotherapy to immunotherapy and targeted therapy, aiming to improve the anti-tumor efficacy and reduce the toxic side effects of treatment [3,4].

Invariant natural killer T (iNKT) cells are a specific subset of T cells that mainly express a constant T cell receptor (TCR) α-chain and a limited TCR β-chain in the human body. iNKT cells are rapidly activated after recognizing lipid and glycolipid antigens presented by CD1d molecules on the surface of antigen-presenting cells (APCs) or exogenous α-Galactosylceramide (α-GalCer), secreting cytokines such as TNF-α, IFN-γ, and IL-4 to exert immune functions [5,6,7,8].

As for the correlation between iNKT cells and malignant tumors, some studies showed that the frequency of circulating iNKT cells was significantly decreased in cancer patients, but Ascui G [9] had reached the opposite conclusion. At the same time, several clinical studies [10,11,12,13,14,15,16,17,18,19,20,21,22,23,24,25,26,27,28] have already explored the efficacy of iNKT cell-related therapies on cancer. A meta-analysis summarizes the findings of multiple studies by integrating evidence from existing studies, and it provides more precise and reliable synthesized conclusions through a series of scientific analyses. Therefore, in order to further explore the correlation between iNKT cells and malignant tumors, this meta-analysis was conducted through three core aspects: firstly, the frequency of circulating iNKT cells in healthy controls and cancer patients was compared. At the same time, we also analyzed the frequency of iNKT cells in tumor tissues and non-tumor tissues. Secondly, the impact of iNKT cells on the prognosis of cancer patients was analyzed. Finally, we evaluated the therapeutic effects of applying iNKT cell-related therapies on cancer.

## 2. Methods

This study followed PRISMA guidelines [29] for meta-analysis (Supplementary Table S1). At the same time, this study has been registered in PROSPERO (Registration number: CRD420251133139).

### 2.1. Search Strategy

PubMed, Web of Science, Embase, and Cochrane Library databases were used to search for relevant studies on iNKT and cancer (involving solid tumors and hematological tumors). The search was conducted by combining MeSH and entry terms related to iNKT cells and cancer. The types of cancer include carcinoma, neoplasm, leukemia, lymphoma, adenocarcinoma, cholangiocarcinoma, glioma, sarcoma, myeloma, and neuroblastoma. The retrieval period was set from the database establishment to August 2025. The references of the relevant studies were also reviewed to find the eligible studies.

### 2.2. Inclusion and Exclusion Criteria

The meta-analysis was required to meet the following criteria: (1) this study involved patients with solid tumors or hematological tumors; (2) the included studies need to include one of the following endpoints: the frequency of iNKT cells in the peripheral blood of healthy controls and cancer patients, as well as in non-tumor and tumor tissues; the impact of iNKT cells on overall survival (OS) and recurrence-free survival (RFS); (3) the objective response rate (ORR) and disease control rate (DCR) of cancer patients without any standard treatment methods by using iNKT cell-related therapies; the patients had not received chemotherapy or radiotherapy in the past 4–6 weeks. The exclusion criteria for this meta-analysis were (1) patients who have received bone marrow transplants; (2) case reports, bioinformatics analysis, conference abstracts, letters, short communications, reviews, commentaries, and animal experiments; (3) studies unable to obtain original data; (4) language was restricted to English.

### 2.3. Data Extraction and Quality Assessment

Two investigators individually reviewed the titles and abstracts to exclude duplicated and the irrelevant literature. Subsequently, the full-text articles were reviewed to screen out qualified studies. The following data were extracted from the clinical trials: first author, publication year, country, sample size, age, gender, duration of expansion, cytokines for expansion, total dose, frequency of infusion, follow-up time, and endpoint. If the included studies only used images to represent the relevant data, we would extract the data with the help of WebPlotDigitizer (https://automeris.io/ (accessed on 9 December 2025). In addition, we used the nine-star Newcastle–Ottawa scale (NOS) [30] to evaluate the quality of observational studies from three aspects: study population selection, component comparability, and measurement of exposure factors, with a score ≤ 4 indicating low quality and a score ≥ 7 indicating high quality. As for clinical trials, we used methodological index for non-randomized studies (MINORS) items [31] for quality assessment. The first eight items were used for studies without control groups, with a maximum score of 16. The last four items, together with the first eight items, were used for studies with control groups, with a maximum score of 24. A score of ≥8 or ≥12 indicated satisfied quality. Any differences were resolved by the third investigator.

### 2.4. Statistical Analysis

Stata 14.0 software was used for statistical analysis. Continuous variables were expressed by using weighted mean difference (WMD) or standardized mean difference (SMD) and their 95% confidence intervals (CI). We calculated WMD for the same measurements and SMD for different measurements. The data were expressed as mean ± standard deviation. If the study only provided the median and quartile (or range), we transformed the data according to Wan’s method [32] to obtain the mean ± standard deviation. Some studies may report multiple types of cancer; in order to avoid the duplication of control groups, we combined the mean ± standard deviation in different cancer types in the same study according to the formula provided in the Cochrane Handbook [33]. We obtained the hazard ratio (HR) and its 95% CI from the text or images in the included studies to analyze the association between iNKT cells and the prognosis of cancer patients (OS and RFS). At the same time, we also evaluated therapeutic effect of applying iNKT cell-related therapies on cancer (ORR and DCR). I^2^ was used to assess the heterogeneity among studies. The random effect model was used if the heterogeneity among studies was significant (I^2^ > 50%); otherwise, the fixed effect model was used. Subgroup analysis was performed based on cancer type, with different iNKT cell-related therapies. Publication bias was assessed by funnel plot and Egger’s test. If the funnel plot was asymmetric and the *p*-value of Egger’s test < 0.05, we used the trim-and-fill method to correct the publication bias and recalculate the pooled statistic. Due to the relatively small number of studies and high heterogeneity between studies, we applied the Hartung–Knapp–Sidik–Jonkman (HKSJ) random-effects model to adjust in our analysis [34]. Furthermore, the leave-one-out method was used for sensitivity analysis. A *p*-value < 0.05 was considered statistically significant.

## 3. Results

### 3.1. Literature Search

Based on the search strategy, a total of 25,975 relevant articles were obtained, and 8685 duplicated articles were excluded. A total of 16,335 articles were excluded based on title and abstract. After reviewing the full text, 889 articles were excluded according to the inclusion and exclusion criteria. Finally, 62 articles were included in this meta-analysis. The literature screening process is shown in Figure 1.

### 3.2. The Basic Characteristics of the Included Studies

A total of 62 articles were included in this meta-analysis, which included 53 observational studies and nine clinical trials. There were a total of 20 articles from Europe, three from Oceania, 11 from the Americas, and 28 from Asia. A total of 1076 healthy controls and 2453 cancer patients were included in this study. The solid tumors involved in this study were lung cancer, esophageal cancer, gastric cancer, hepatocellular carcinoma, prostate cancer, colorectal cancer, breast cancer, cervical cancer, renal cell carcinoma, melanoma, glioma, neuroblastoma, head and neck squamous cell carcinoma, malignant mesothelioma, etc. The hematological tumors involved in this study were leukemia, myeloma, lymphoma, etc. The basic information of the included studies is shown in Table 1 and Table 2.

### 3.3. Quality Assessment

NOS and MINORS items were used to evaluate the quality of the included studies. Among the 53 observational studies, 14 were high quality (NOS scores ≥ 7) and 39 were moderate quality (NOS scores ranged from four to six). The nine clinical trials did not have control groups, so the first eight items were used for assessment, and the MINORS scores ranged from nine to 12, indicating that the methodological quality of the clinical trials were satisfactory. The quality assessment of the included studies is shown in Table 1 and Table 2.

### 3.4. Meta-Analysis and Subgroup Analysis

#### 3.4.1. The Frequency of Circulating iNKT Cells in Cancer Patients

##### The Frequency of iNKT Cells in Circulating T Cells

A total of 19 studies reported the frequency of iNKT cells in circulating T cells of healthy controls and cancer patients, including 13 studies [9,38,39,44,47,55,58,66,75,76,81,83,84] involving solid tumors and six studies [56,57,61,63,67,80] involving hematological tumors. The meta-analysis showed that the frequency of iNKT cells in circulating T cells was significantly higher in healthy controls than in cancer patients (WMD = 0.074, 95% CI: 0.040–0.109, *p* < 0.001; I^2^ = 85.2%, *p* < 0.001). Subgroup analysis based on cancer type showed the frequency of iNKT cells in circulating T cells was significantly higher in healthy controls than solid (WMD = 0.089, 95% CI: 0.031–0.147, *p* = 0.003; I^2^ = 87.6%, *p* < 0.001) and hematological (WMD = 0.053, 95% CI: 0.010–0.095, *p* = 0.015; I^2^ = 79.7%, *p* < 0.001) tumor patients. See Figure 2.

Due to the high heterogeneity between studies, we applied the HKSJ adjustment to the results. After applying HKSJ adjustment to the results, higher frequency of iNKT cells in circulating T cells was observed in healthy controls than in solid tumor patients. However, no significant difference was observed between healthy controls and hematological tumor patients (Supplementary Figure S1). In addition, we also performed subgroup analysis based on specific cancer type. The results showed that the frequency of iNKT cells in circulating T cells was higher in healthy controls than in head and neck squamous cell carcinoma and gynecologic malignancies (*p* < 0.001, *p* = 0.003, Supplementary Figure S2).

##### The Frequency of iNKT Cells in Circulating Mononuclear Cells

A total of 16 studies reported the frequency of iNKT cells in circulating mononuclear cells of healthy controls and cancer patients. Among them, there were six studies [40,43,53,62,70,73] involving solid tumors, nine studies [37,42,48,52,60,64,65,72,79] involving hematological tumors, and one study [35] involving solid and hematological tumors. The meta-analysis showed that the frequency of iNKT cells in circulating mononuclear cells was significantly higher in healthy controls than in cancer patients (WMD = 0.053, 95% CI: 0.019–0.086, *p* = 0.002; I^2^ = 80.9%, *p* < 0.001). Subgroup analysis based on cancer type showed that the frequency of iNKT cells in circulating mononuclear cells of healthy controls was similar to solid tumor patients (WMD = 0.033, 95% CI: −0.021–0.087, *p* = 0.234; I^2^ = 63%, *p* = 0.013), but significantly higher than hematological tumor patients (WMD = 0.076, 95% CI: 0.019–0.132, *p* = 0.008; I^2^ = 86.6%, *p* < 0.001). See Figure 3.

Similarly, we also performed HKSJ adjustment to the results. However, no significant difference was observed between healthy controls and patients with solid tumors or hematological malignancies (Supplementary Figure S3). In addition, we also performed subgroup analysis based on specific cancer type. The results showed that, compared to healthy controls, the frequency of iNKT cells in circulating monouclear cells was significantly decreased in lymphoma patients (*p* = 0.018, Supplementary Figure S4).

##### The Frequency of iNKT Cells in Circulating Lymphocytes

A total of seven studies reported the frequency of iNKT cells in circulating lymphocytes of healthy controls and cancer patients, including five studies [36,41,59,77,82] involving solid tumors and two studies [54,57] involving hematological tumors. The meta-analysis showed that the frequency of iNKT cells in circulating lymphocytes of healthy controls was similar to cancer patients (WMD = 0.018, 95% CI: −0.014–0.050, *p* = 0.262; I^2^ = 85.7%, *p* < 0.001). Subgroup analysis based on cancer type showed that the frequency of iNKT cells in circulating lymphocytes of healthy controls was similar to solid (WMD = 0.020, 95% CI: −0.026–0.065, *p* = 0.398; I^2^ = 89.9%, *p* < 0.001) and hematological (WMD = 0.021, 95% CI: −0.012–0.055, *p* = 0.207; I^2^ = 43.9%, *p* = 0.182) tumor patients. See Figure 4.

The results of HKSJ adjustment showed no difference in the frequency of iNKT cells in circulating lyophocytes between healthy controls and patients with solid tumor or hematological malignancies (Supplementary Figure S5). Further subgroup analysis based on specific cancer type showed that, compared to healthy controls, the frequency of iNKT cells in circulating lymphocytes was decreased in lung caner. (*p* < 0.001, Supplementary Figure S6).

##### The Number of Circulating iNKT Cells

A total of 12 studies reported the number of circulating iNKT cells of healthy controls and cancer patients, including seven studies [12,39,43,45,59,69,77] involving solid tumors and five studies [50,52,54,61,78] involving hematological tumors. The meta-analysis showed that the number of circulating iNKT cells was significantly higher in healthy controls than in cancer patients (SMD = 0.632, 95% CI: 0.271–0.994, *p* = 0.001; I^2^ = 82.2%, *p* < 0.001). Subgroup analysis based on cancer type showed that the number of circulating iNKT cells was higher in healthy controls than in solid (SMD = 0.700, 95% CI: 0.155–1.244, *p* = 0.012; I^2^ = 82.7%, *p* < 0.001) and hematological (SMD = 0.533, 95% CI: 0.031–1.036, *p* = 0.037; I^2^ = 83.1%, *p* < 0.001) tumor patients. See Figure 5.

However, the results of HKSJ adjustment showed that there was no significant difference between healthy controls and patients with solid tumor or hematological malignancies (Supplementary Figure S7). In addition, the subgroup analysis showed that compared to healthy controls, the number of circulating iNKT cells was lower in lung cancer patients (*p* < 0.001, Supplementary Figure S8).

#### 3.4.2. The Frequency of iNKT Cells to T Cells in Tissues

A total of five studies reported the frequency of iNKT cells to T cells in non-tumor and tumor tissues, including one study [71] involving colorectal cancer, three studies [38,58,68] involving hepatocellular carcinoma, and one study [81] involving ovarian cancer. The meta-analysis showed the frequency of iNKT cells to T cells was significantly higher in non-tumor tissues than in tumor tissues (WMD = 0.093, 95% CI: 0.004–0.181, *p* = 0.040; I^2^ = 39.1%, *p* = 0.160). See Figure 6. The subgroup analysis showed that the frequency of iNKT cells was lower in hepatocellular carcinoma tissues than non-tumor tissues. (*p* = 0.025, see Supplementary Figure S9).

#### 3.4.3. The Impact of iNKT Cells on Prognosis

##### Overall Survival (OS)

A total of nine studies reported the association between the frequency of iNKT cells and OS in cancer patients, including four studies [20,49,51,76] involving tumor tissues and five studies [9,45,46,74,81] involving peripheral blood. The meta-analysis showed that the higher frequency of iNKT cells in cancer patients was associated with improved OS (HR = 0.568, 95% CI: 0.466–0.693, *p* < 0.001; I^2^ = 26.4%, *p* = 0.209). Subgroup analysis based on cancer type showed that a higher frequency of iNKT cells in tumor tissues (HR = 0.476, 95% CI: 0.350–0.647, *p* < 0.001; I^2^ = 47%, *p* = 0.129) and peripheral blood (HR = 0.645, 95% CI: 0.497–0.838, *p* = 0.001; I^2^ = 0.0%, *p* = 0.555) may be associated with improved OS. See Figure 7. Further subgroup analysis based on specific cancer types showed that the higher frequency of iNKT cells in patients with digestive system malignancies or neuroblastoma were associated with improved OS (*p* = 0.003, *p* < 0.001, see Supplementary Figure S10).

##### Relapse-Free Survival (RFS)

A total of four studies reported the association between the frequency of iNKT cells and RFS in cancer patients, including two studies [49,51] involving tumor tissues and two studies [22,45] involving peripheral blood. The meta-analysis showed that a higher frequency of iNKT cells in cancer patients could be associated with improved RFS (HR = 0.374, 95% CI: 0.161–0.868, *p* = 0.022; I^2^ = 59.5%, *p* = 0.060). However, subgroup analysis based on type showed that a higher frequency of iNKT cells in tumor tissues (HR = 0.312, 95% CI: 0.072–1.350, *p* = 0.119; I^2^ = 72.9%, *p* = 0.055) and peripheral blood (HR = 0.275, 95% CI: 0.023–3.253, *p* = 0.306; I^2^ = 72.9%, *p* = 0.055) was not significantly associated with improved RFS. See Figure 8. These findings were consistent with the HKSJ-adjusted results (Supplementary Figure S11). After subgroup analysis based on different cancer types, the results showed that the frequency of iNKT cells was associated with improved RFS in digestive system malignancies (*p* = 0.047, Supplementary Figure S12).

#### 3.4.4. The Efficacy of iNKT Cell-Related Therapies

##### Objective Response Rate (ORR)

A total of seven studies, with a combined total of 119 cancer patients, reported ORR in solid tumor patients treated with α-GalCer, α-GalCer-Pulsed APC, iNKT cells, or iNKT cells combined with α-GalCer-Pulsed APC. Among them, one study [12] involving α-GalCer, four studies [13,14,17,18] involving α-GalCer-Pulsed APC, one study involving iNKT cells [16], and one study [15] involving iNKT cells combined with α-GalCer-Pulsed APC. The meta-analysis showed that the ORR of iNKT cell-related therapies was 3% (95% CI: 0.00–0.11, *p* = 0.04; I^2^ = 38.34%, *p* = 0.14). See Figure 9.

In further subgroup anlaysis by specific cancer types, the results showed that the ORR of iNKT cell-related therapies in head and neck squamous cell carcinoma patients was 22%, and was only 1% in non-small cell lung cancer patients (Supplementary Figure S13). In addition, we also performed subgroup analysis based on different iNKT cell-related therapies (Supplementary Figure S14).

##### Disease Control Rate (DCR)

A total of nine studies, with a combined total of 117 cancer patients, reported DCR in solid tumor patients treated with α-GalCer, α-GalCer-Pulsed APC, iNKT cells, or iNKT cells combined with α-GalCer-Pulsed APC. Among them, there was one study [12] involving α-GalCer, five studies [13,14,17,18] involving α-GalCer-Pulsed APC, three studies involving iNKT cells [10,11,16], and one study [15] involving iNKT cells combined with α-GalCer-Pulsed APC. The meta-analysis showed that the DCR of iNKT cell-related therapies was 49% (95% CI: 0.38–0.61, *p* = 0.03; I^2^ = 31.26%, *p* = 0.17). See Figure 10.

After further subgroup anaysis based on cancer type, we found that the DCR of iNKT cell-related therapies was 77% in head and neck squamous cell carcinoma and 48% in non-small cell lung cancer (Supplementary Figure S15). Subgroup analysis based on different iNKT cell-related therapies revealed that the DCR of α-GalCer-Pulsed APC and iNKT cells was 49% and 48%, respectively (see Supplementary Figure S16).

### 3.5. Publication Bias

Publication bias was detected by funnel plot and Egger test. The results showed that publication bias existed in the frequency of iNKT cells in circulating T cells and in circulating mononuclear cells (the funnel plot was asymmetric, and the *p*-value of the Egger test < 0.05). No publication bias was observed for the other endpoints. (See Supplementary Figures S17–S25). Therefore, the trim-and-fill method was used to correct for funnel plot asymmetry caused by the publication bias. The redrawn funnel plot was symmetrical after adding the six missing hypothetical studies. The recalculated pooled statistic showed that the frequency of iNKT cells in circulating T cells was significantly higher in healthy controls than in cancer patients (WMD = 0.022, 95% CI: 0.012–0.032, *p* < 0.001). (Supplementary Figure S17). In addition, after adding the four missing studies, the recalculated pooled statistic indicated that the frequency of iNKT cells in circulating mononuclear cells was higher in healthy controls than in cancer patients (WMD = 0.005, 95% CI: 0.001–0.009, *p* = 0.011), which was consistent with the original results (Supplementary Figure S18).

### 3.6. Heterogeneity Analysis

High heterogeneity was observed in the analysis of iNKT cells frequency in cancer patients and healthy controls (I^2^ > 50%). We performed the subgroup analysis based on specific cancer types to identify the sources of heterogeneity. The results showed that the heterogeneity among the studies had decreased compared to before (Supplementary Figures S2, S4, S6 and S8). Similarly, in the analysis of iNKT cell-related therapies and the outcomes of tumor patients, we conducted subgroup analysis according to iNKT cell-related therapies, and reduction in heterogeneity was observed (Supplementary Figures S14 and S16). Therefore, the above results indicated that the cancer type and different iNKT cell therapy could be the source of heterogeneity.

### 3.7. Sensitivity Analysis

To assess the reliability and robustness of the analytical results in this study, we performed a sensitivity analysis using the leave-one-out method. The results showed that sequential exclusion of each individual study did not significantly affect the overall findings, indicating that the results of our study are relatively robust and stable (see Supplementary Figures S26–S29).

## 4. Discussion

This meta-analysis analyzed the frequency of iNKT cells in healthy control and cancer patients, involving peripheral blood and tissues. It also analyzed the association between the frequency of iNKT cells and the prognosis of cancer patients. Finally, it assessed the efficacy of the iNKT cell-related therapies on cancer. The meta-analysis showed the that the circulating iNKT cells were significantly higher in healthy controls than in cancer patients, as well as the frequency of iNKT cells in circulating T cells and circulating mononuclear cells. However, they were similar in the frequency of iNKT cells in circulating lymphocytes. In addition, the frequency of iNKT cells in circulating T cells was significantly higher in non-tumor tissues than in tumor tissues. Regarding the prognosis of cancer patients, we found that a higher frequency of iNKT cells in cancer patients was associated with improved OS/RFS. As for the efficacy of the cancer, we found that the ORR of iNKT cell-related therapies was 3% and the DCR was 49%.

Several studies have shown that iNKT cells in cancer patients often exhibit the dual abnormalities of “decreased quantity” and “defective function”, and this phenomenon is common in various malignant tumors. By comparing 47 patients with head and neck squamous cell carcinoma with 33 healthy controls, Molling JW [45] found that the number of circulating iNKT cells in the patients was significantly decreased. The phenomenon of reduced iNKT cell numbers has been verified in patients with various malignant tumors, such as breast cancer [40], upper gastrointestinal cancers [75], lung cancer [59,70], hepatocellular carcinoma [58], melanoma [36], leukemia [37,54,61], lymphoma [48,72] and myeloma [57,78]. At the functional level, iNKT cells generally have a diminished proliferative response capacity and elevated expression levels of markers related to depletion/inhibition on the cell surface [48,58,70], suggesting that the iNKT cells are in a state of functional inhibition. It is worth noting that Lattanzi G [71] discovered in the research on colorectal cancer that despite the enrichment of iNKT cells in tumor tissues, the expression level of IFN-γ was decreased in iNKT cells, while the expression of PD-1, TIGIT, and TIM-3 was up-regulated. When exogenous α-GalCer was administered, it could re-activate the anti-tumor immunity mediated for iNKT cells. The above studies indicated that there were quantitative deficiencies or dysfunctions of iNKT cells in cancer patients, which may be the vital reason for the weakening anti-tumor immune response in the body. The meta-analysis showed that the frequency of iNKT cells in the peripheral blood and tissues of the healthy controls were significantly higher than cancer group. Moreover, the higher frequency of iNKT cells could be associated with improved OS and RFS.

Animal experiments have shown that iNKT cells are defective in tumor-bearing mice, which is similar to humans. When given exogenous stimulants (IL-2 and α-GalCer), this trend can be reversed and anti-tumor immunity can be promoted [23]. Furthermore, α-GalCer can also enhance the anti-tumor effects of iNKT cells [24,72]. If the α-GalCer is loaded on the surface of APC to act as the specific activation carrier for iNKT cells, it can further enhance the anti-tumor effect of iNKT cells [25]. At the same time, adoptive immunotherapy with iNKT cells has also exhibited a powerful anti-tumor effect [26,27]. Based on the therapeutic potential of iNKT cell-related therapies in tumor-bearing mice, this meta-analysis included relevant clinical studies for further exploration. It should be noted that the studies included in this meta-analysis were advanced and involved recurrent malignant tumor patients without any standard treatment methods. Although iNKT cell-related therapies have demonstrated anti-tumor effects, these effects were mainly manifested in the DCR. For patients with operable malignant tumors, Yamasaki, K [19] found that the ORR of iNKT cells combined with α-GalCer-Pulsed APC before salvage surgery was approximately 50%. However, Iyoda, T [21] observed that the RFS in postoperative early-stage lung cancer patients treated with α-GalCer-Pulsed APC was similar to that in the standard treatment group. This suggests that iNKT cell-related therapies could be an alternative treatment option for patients with malignant tumors. However, due to the small number of patients, the efficacy of these therapies for malignant tumors still need to be explored.

This meta-analysis has some limitations: Firstly, some studies did not directly provide the original data (e.g., mean ± standard deviation, etc.) required for meta-analysis, so we used the WebPlotDigitizer (https://automeris.io/) and related formulas to extract and transform the graphs or data from the included studies [32,33]. Although these methods are commonly tools for dealing with missing data in current meta-analyses, the subjective judgments in the data extraction process and the formula conversion approximations may still lead to some deviation in the final data from the original data. Some methodological limitations should also be acknowledged, including a small number of studies for individual cancer types, substantial heterogeneity for some outcomes, publication bias in certain analysis, and the moderate methodological quality of most included observational studies according to the NOS. These factors may have introduced bias and limited our conclusions. It should also be noted that none of the nine clinical trials included a control group, which limits the interpretation of the clinical outcomes. Finally, since we analyzed different clinical issues, the inclusion criteria were overly broad, which is also one of the limitations of this study.

In conclusion, this meta-analysis showed that the frequency of iNKT cells in cancer patients was significantly reduced in either peripheral blood or tumor tissues. Furthermore, the higher frequency of iNKT cells was closely associated with the prognosis of the cancer patients, specifically manifested as the improvement in OS and RFS.

## Figures and Tables

**Figure 1:**
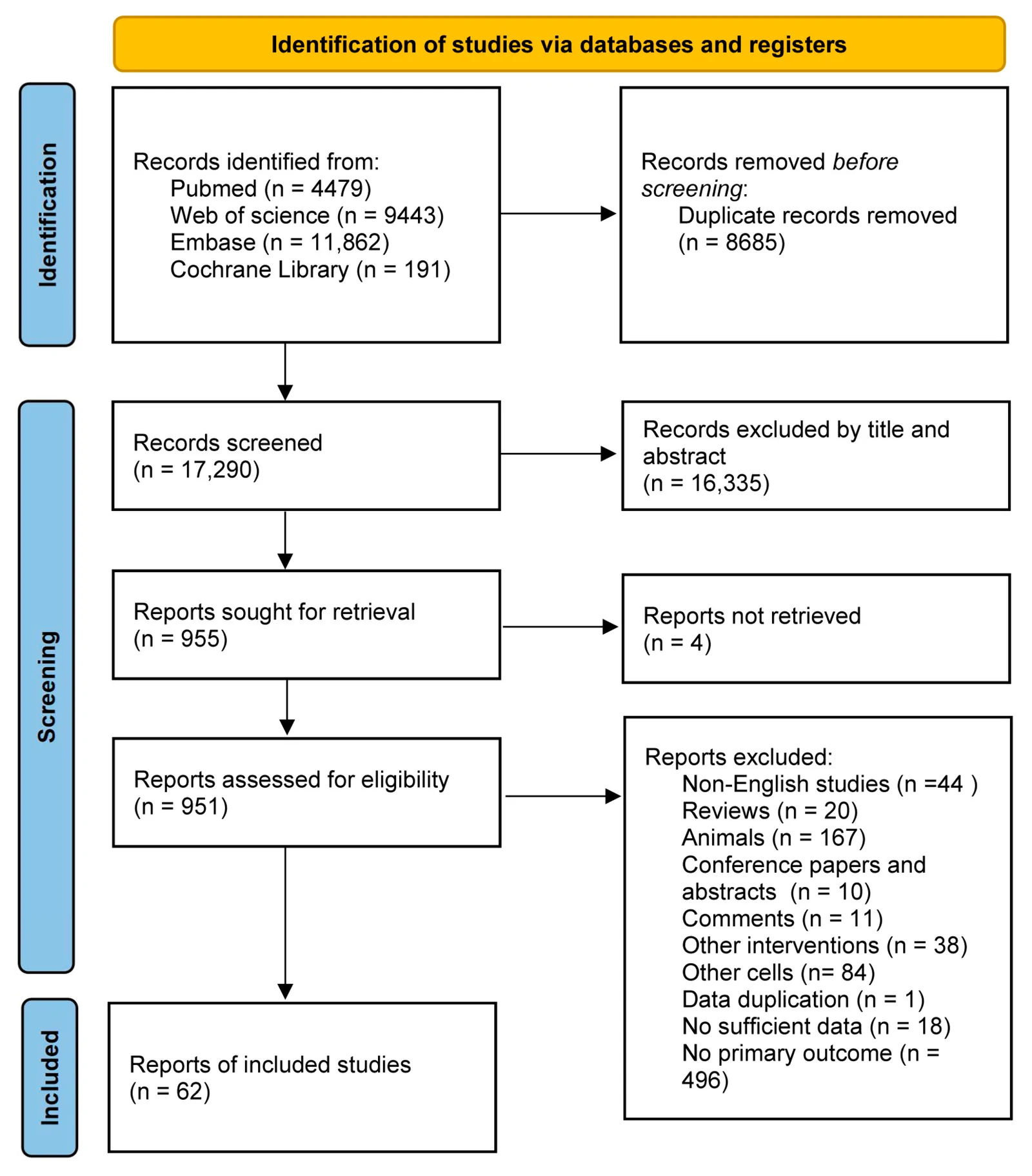
Flow chart of the literature search.

**Figure 2:**
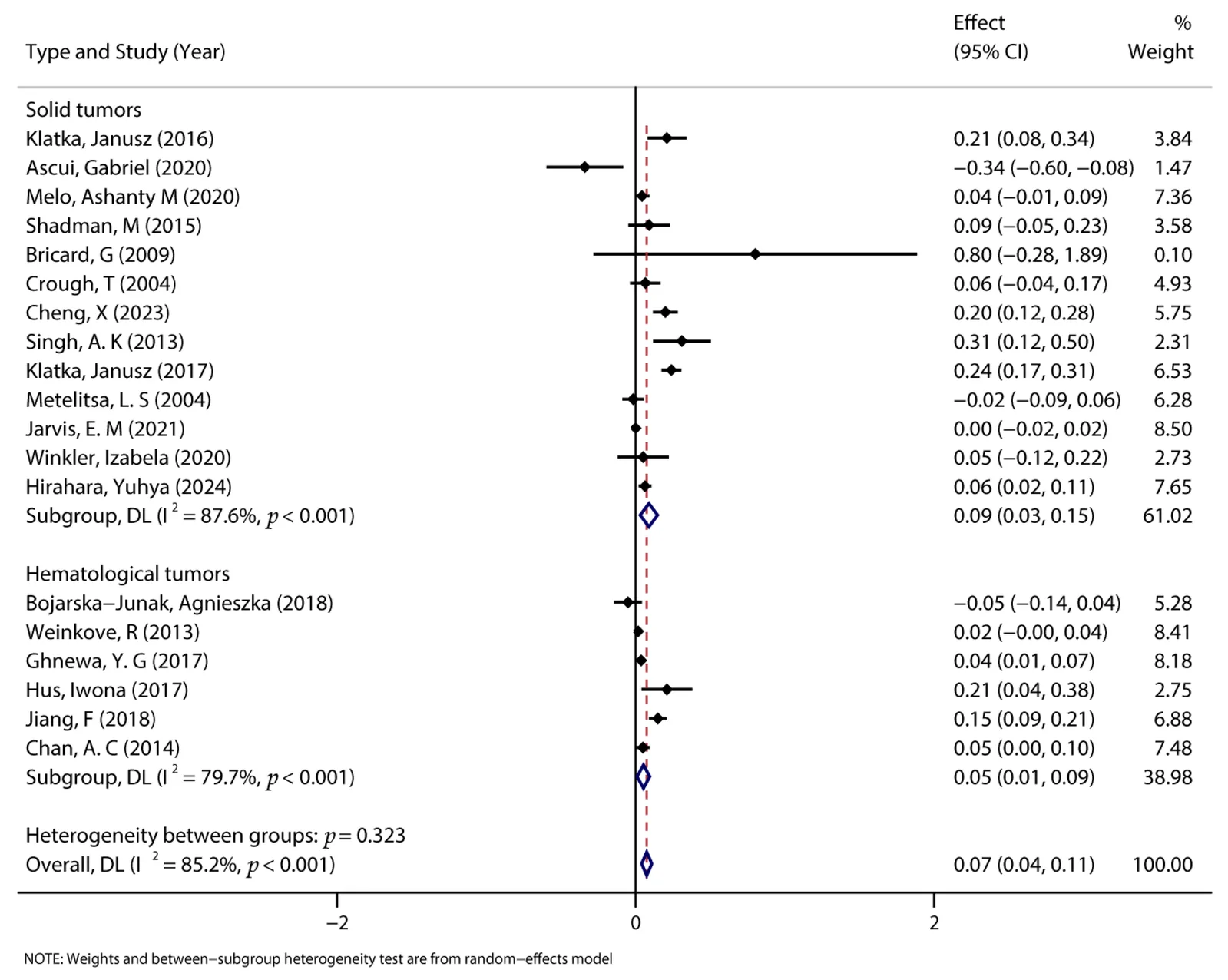
The forest plot showing the frequency of iNKT cells in circulating T cells between healthy controls and cancer patients [9,38,39,44,47,55,56,57,58,61,63,66,67,75,76,80,81,83,84].

**Figure 3:**
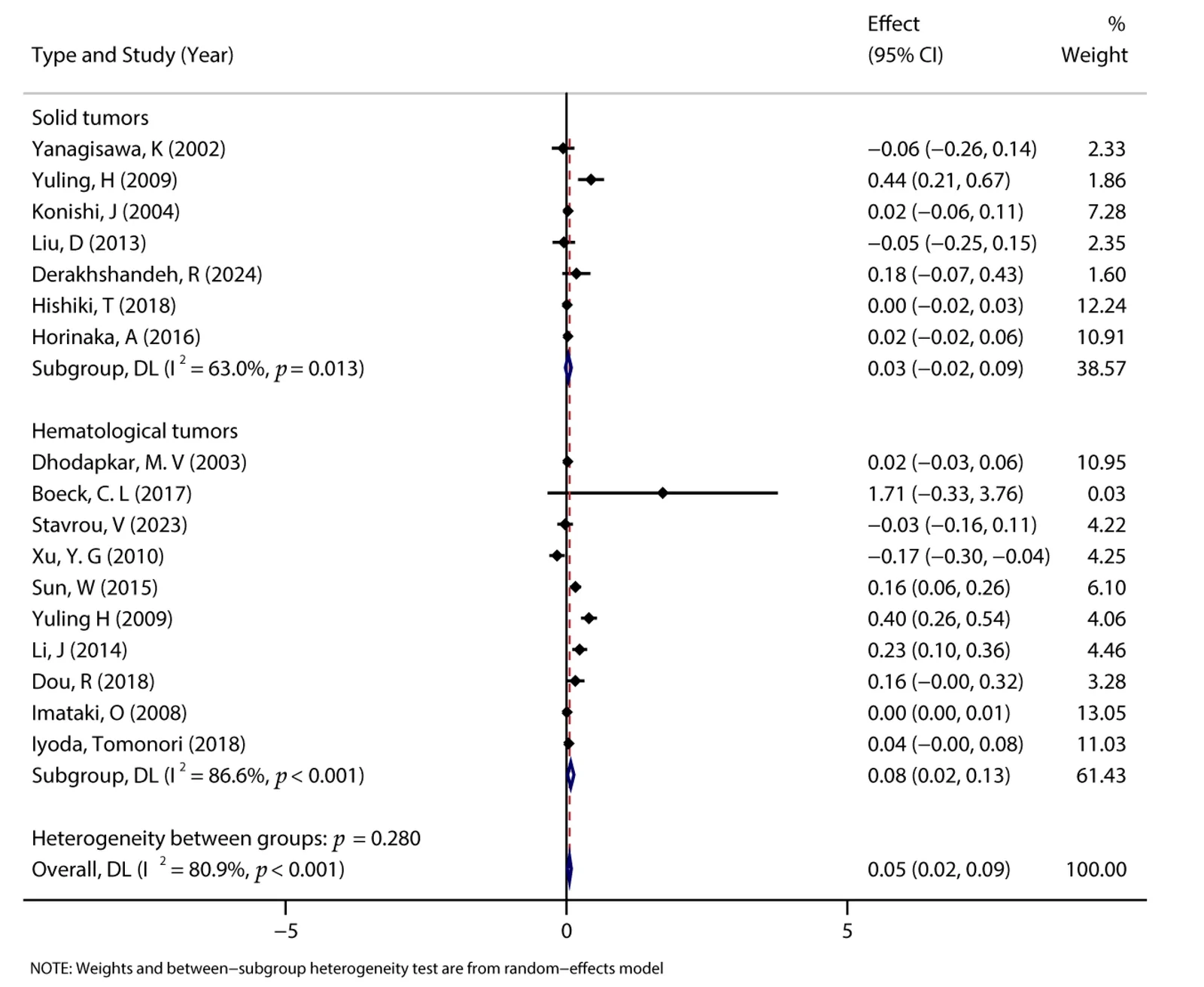
The forest plot showing the frequency of iNKT cells in circulating mononuclear cells between healthy controls and cancer patients [35,37,40,42,43,48,52,53,60,62,64,65,70,72,73,79].

**Figure 4:**
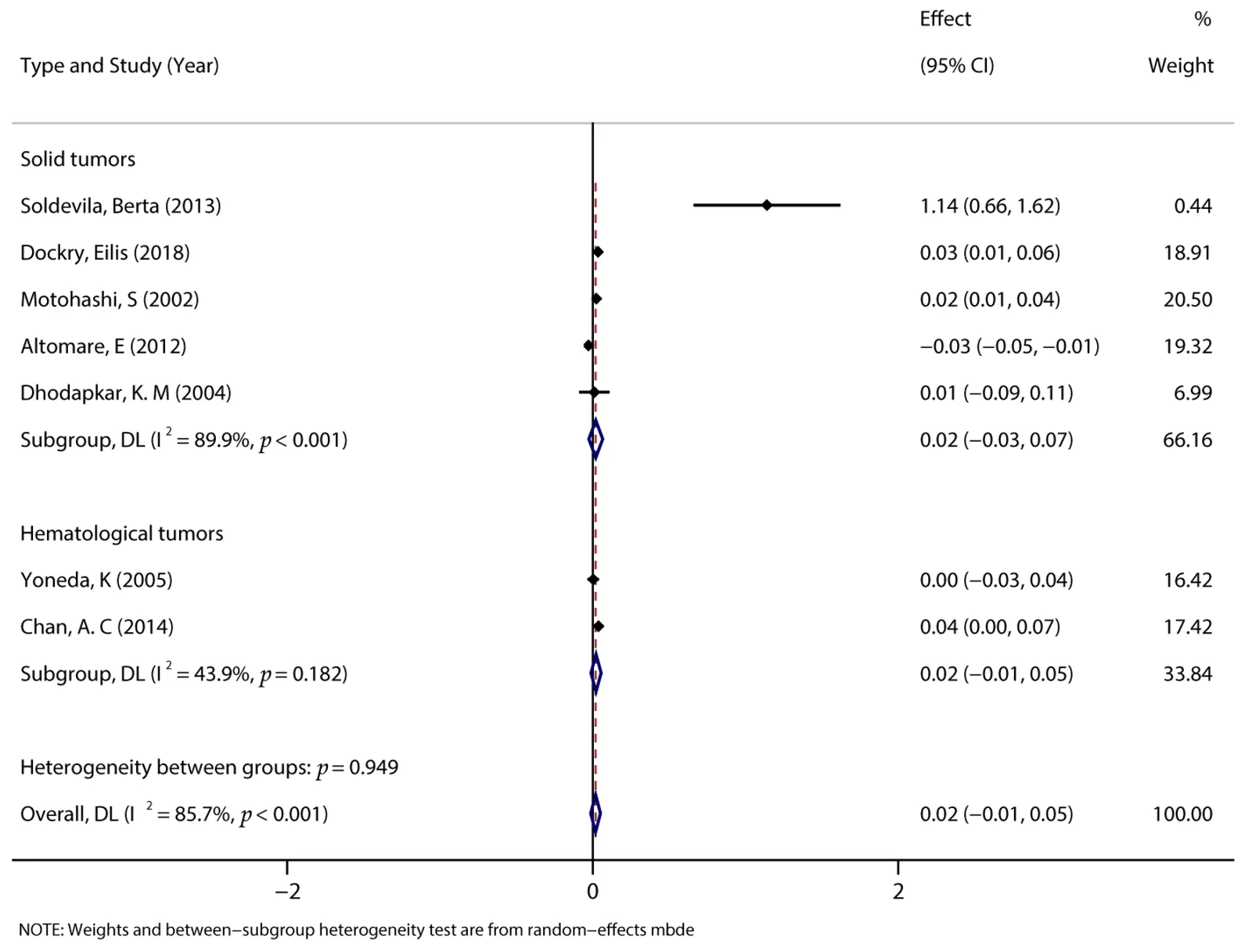
The forest plot showing the frequency of iNKT cells in circulating lymphocytes between healthy controls and cancer patients [36,41,54,57,59,77,82].

**Figure 5:**
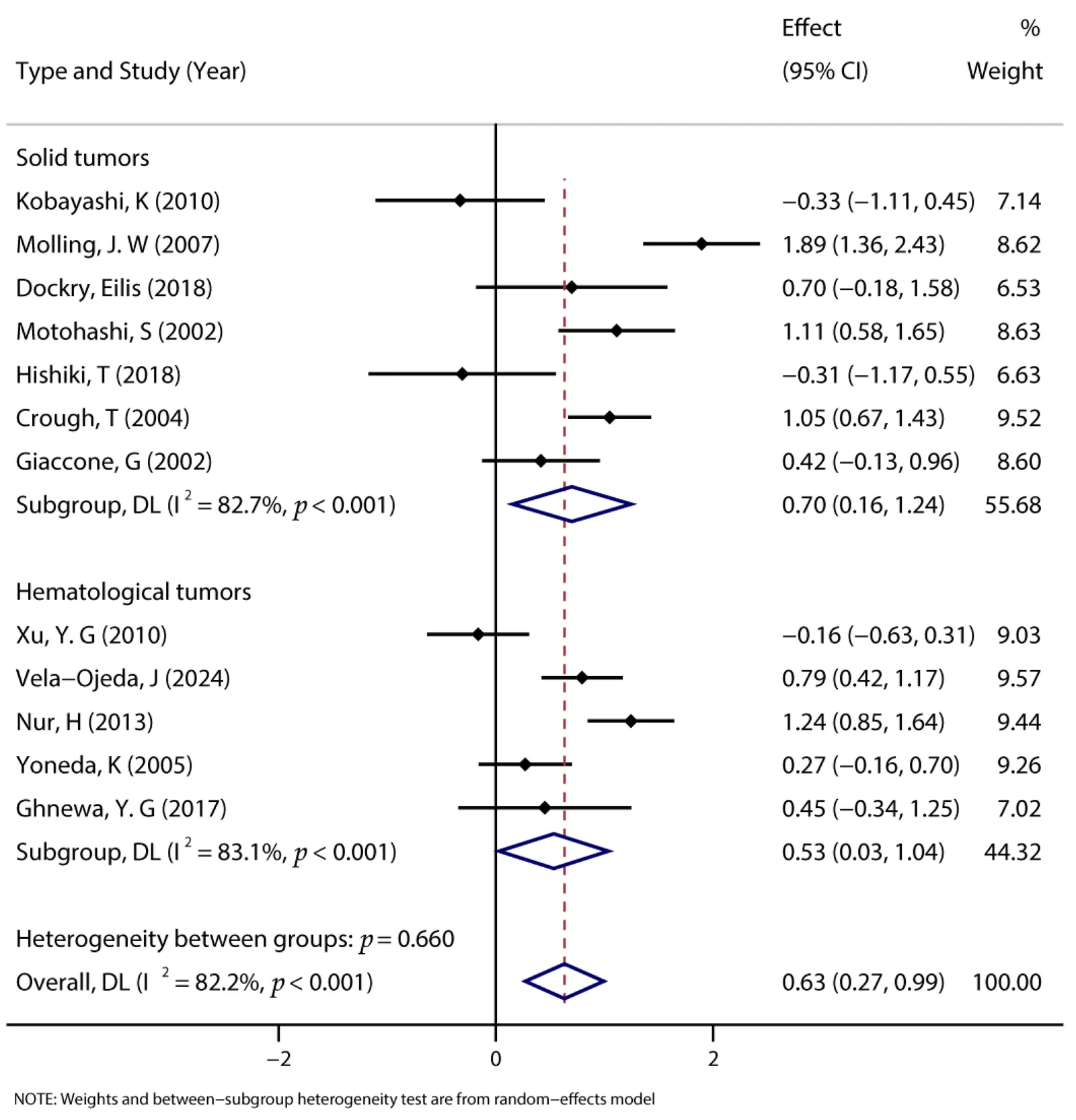
The forest plot showing the number of circulating iNKT cells between healthy controls and cancer patients [12,39,43,45,50,52,54,59,61,69,77,78].

**Figure 6:**
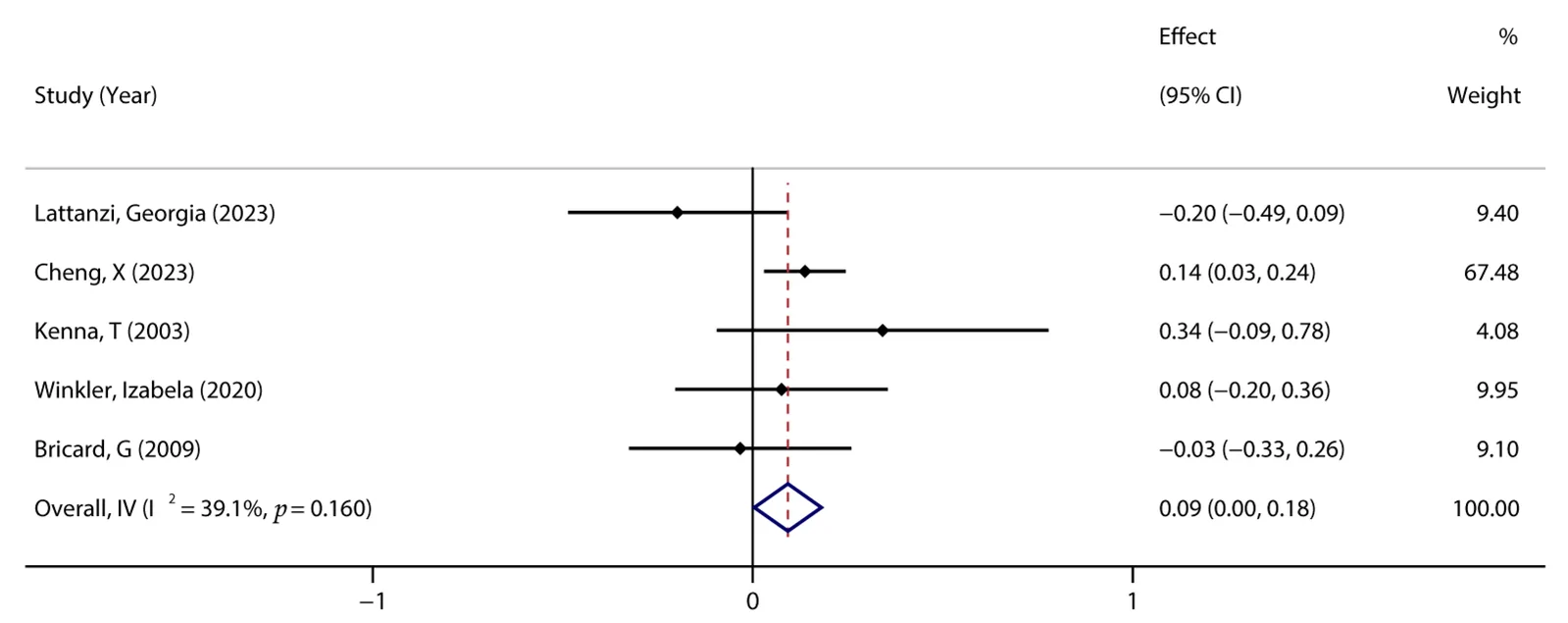
The forest plot showing the frequency of iNKT cells to T cells in non-tumor and tumor tissues [38,58,68,71,81].

**Figure 7:**
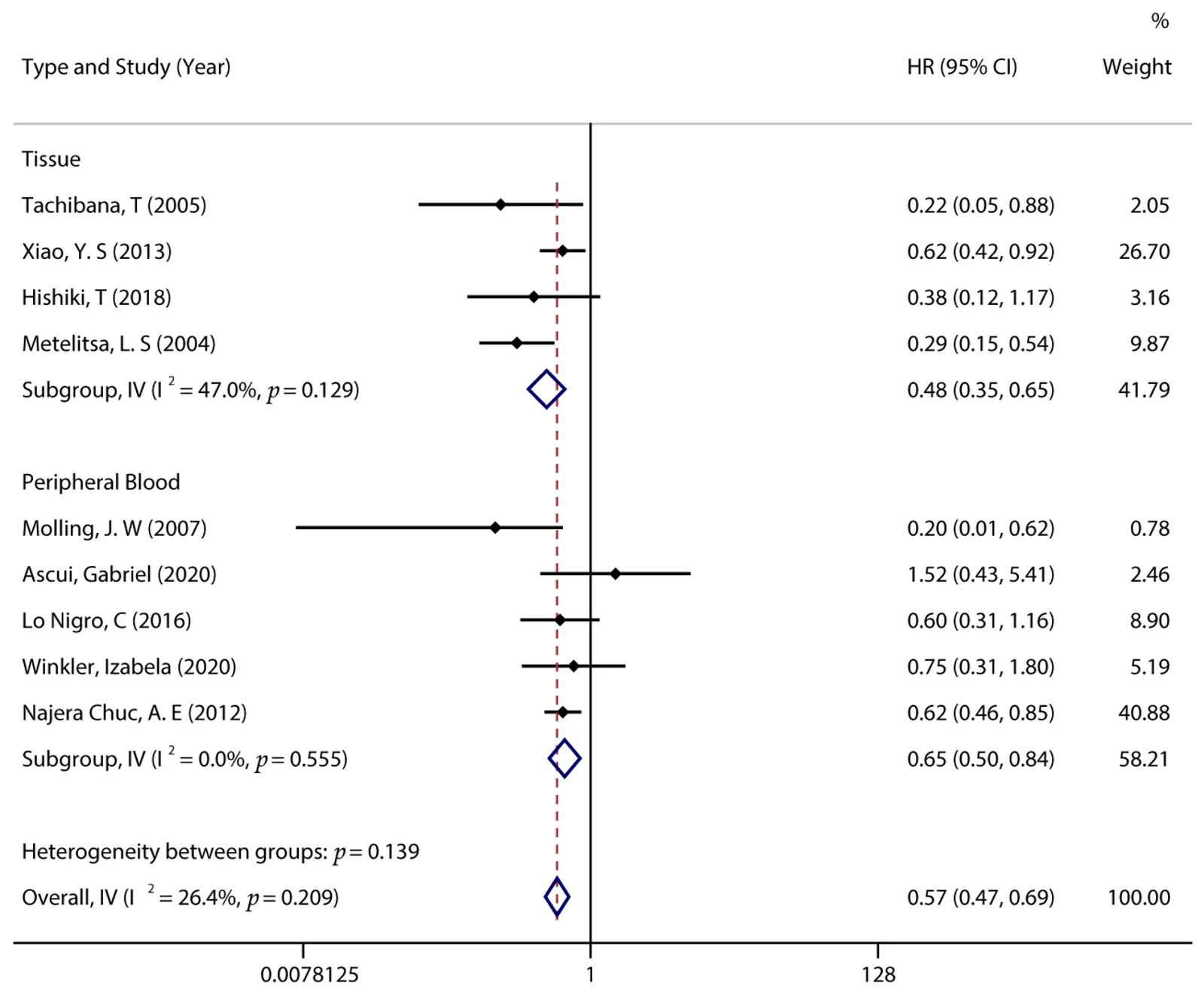
The forest plot showing higher frequency of iNKT cells and overall survival [9,20,45,46,49,51,74,76,81].

**Figure 8:**
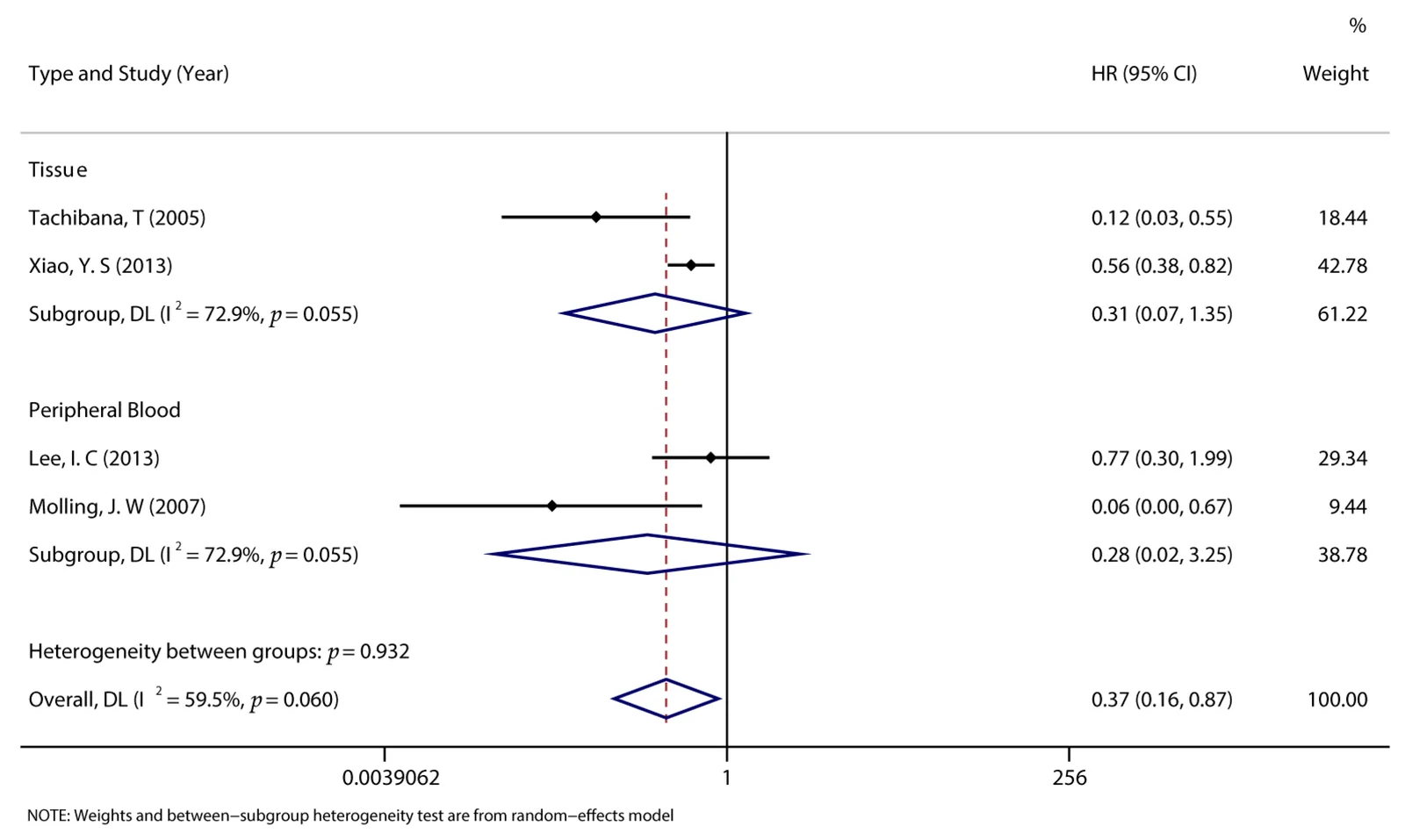
The forest plot showing higher frequency of iNKT cells and relapse-free survival [22,45,49,51].

**Figure 9:**
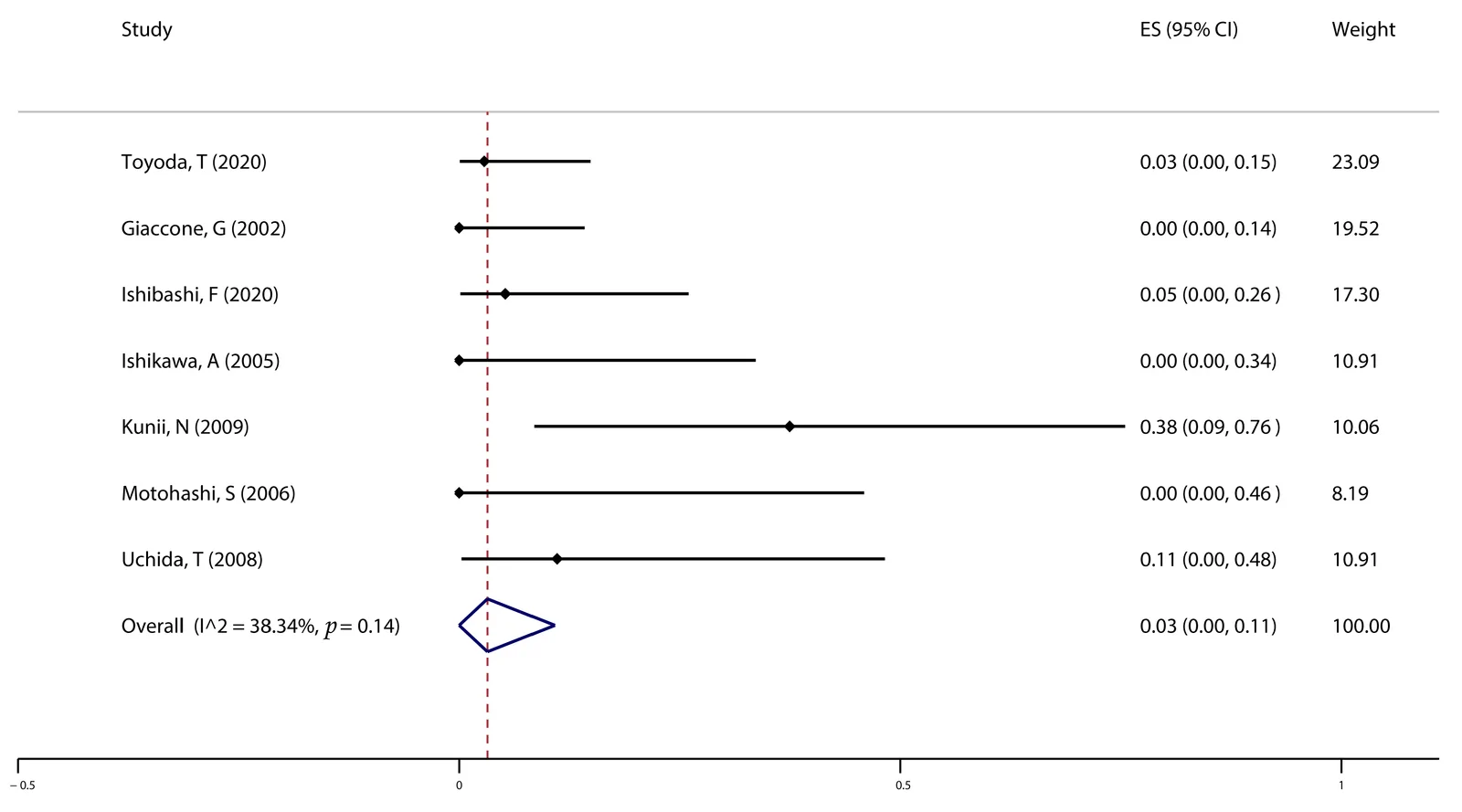
The objective response rate of iNKT cell-related therapies [12,13,14,15,16,17,18].

**Figure 10:**
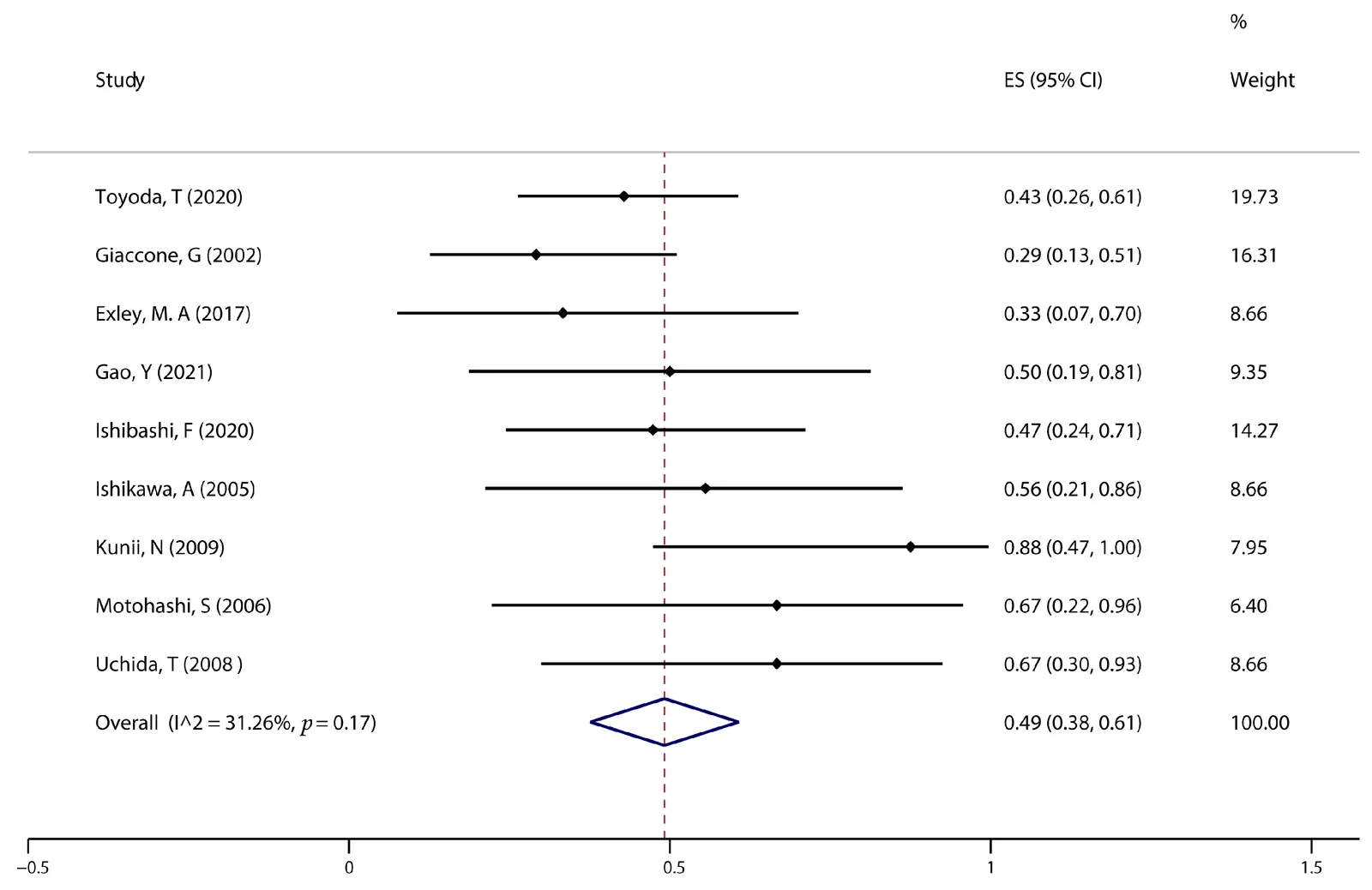
The disease control rate of iNKT cell-related therapies [10,11,12,13,14,15,16,17,18].

**Table 1:** The basic characteristics of observational studies.

Author	Year	Country	Sample Size	Age (Years) (Mean ± Standard Deviation or Median and Range)	Male (%)	iNKT Markers	Endpoint	NOS Score
Ascui, G. [9]	2020	Chile	HC = 23 Gastric cancer, Normal percentages of peripheral iNKT cells = 10, High percentages of peripheral iNKT cells = 11	-	-	Vα24^+^Jα18^+^Vβ11^+^	the frequency of iNKT cells in peripheral blood, OS	5 (3/0/2)
Yuling, H. [35]	2009	China	HC = 6 Hodgkin’s lymphoma = 6 Nasopharyngeal carcinoma = 6	-	-	αGalCer- CD1d tetramer^+^TCRβ^+^	the frequency of iNKT cells in peripheral blood	5 (2/0/3)
Soldevila, B. [36]	2013	Spain	HC = 9, Melanoma = 9	HC = 42.44 ± 16.39, Melanoma = 47.56 ± 11.89	HC = 55.56%, Melanoma = 55.56%	Vα24^+^Jα18^+^	the frequency of iNKT cells in peripheral blood	8 (3/2/3)
Boeck, C.L. [37]	2017	Germany	HC = 9, AML = 23, ALL = 20, CLL = 21	AML = 50 (range: 23–81), ALL = 22 (range: 3–50), CLL = 57 (range: 33–72)	AML = 65.21%, ALL = 60%, CLL = 47.62%	6B11^+^, 6B11^+^CD3^+^ or CD8^+^ or CD161^+^ or Va24^+^	the frequency of iNKT cells in peripheral blood	5 (2/0/3)
Bricard, G. [38]	2009	Switzerland	HC = 15, HCC = 9, Colon carcinoma metastasis = 4, Uveal melanoma metastasis = 4	HCC = 66.33 ± 9.07, Colon carcinoma metastasis = 49.5 ± 14.39, Uveal melanoma metastasis = 67.75 ± 7.27	HCC = 88.89%, Colon carcinoma metastasis = 0%, Uveal melanoma metastasis = 25%	Vα24^+^Vβ11^+^	the frequency of iNKT cells in peripheral blood and tissues	5 (2/0/3)
Crough, T. [39]	2004	Australia	HC = 40, Colorectal cancer = 33, Breast cancer = 10, Melanoma = 17, Lung cancer = 8, Renal cell carcinoma = 10, Other cancers = 31	HC = 34 (19–68), Colorectal cancer = 61 (43–79), Breast cancer = 54 (41–69), Melanoma cancer = 59 (26–83), Lung cancer = 55 (33–72), Renal cell carcinoma = 53 (36–67), Other cancers = 58 (27–72)	HC = 37.5%, Colorectal cancer = 66.67%, Breast cancer = 0%, Melanoma cancer = 82.35%, Lung cancer = 75%, Renal cell carcinoma = 90%, Other cancers = 41.94%	Vα24^+^Vβ11^+^	the frequency of iNKT cells in peripheral blood	6 (3/0/3)
Derakhshandeh, R. [40]	2024	USA	HC = 18, Breast cancer = 30	Breast cancer = 51.5 (20–89)	HC = 0%, Breast cancer = 0%	CD3^+^Vα24^+^Jα18^+^	the frequency of iNKT cells in peripheral blood	6 (3/0/3)
Dhodapkar, K.M. [41]	2004	USA	HC = 9, Glioma = 9	-	-	Vα24^+^Vβ11^+^	the frequency of iNKT cells in peripheral blood	5 (2/0/3)
Dhodapkar, M.V. [42]	2003	USA	HC = 13 Multiple myeloma = 11	-	-	Vα24^+^Vβ11^+^	the frequency of iNKT cells in peripheral blood	5 (2/0/3)
Hishiki, T. [43]	2018	Japan	HC = 11, Neuroblastoma = 6, Hepatoblastoma = 2, Rhabdomyosarcoma = 2	HC = 3.83 (0.75–7.5) Neuroblastoma = 3.29 ± 2.49, Hepatoblastoma = 1.58 ± 0.71, Rhabdomyosarcoma = 0.46 ± 0.41	-	Vα24^+^Vβ11^+^	the frequency of iNKT cells in peripheral blood	7 (3/1/3)
Klatka, J. [44]	2016	Poland	HC = 15 Laryngeal cancer = 50	HC = 61 (43–82) Laryngeal cancer = 60 (45–77)	HC = 80% Laryngeal cancer = 80%	CD3^+^anti-iNKT^+^	the frequency of iNKT cells in peripheral blood	9 (4/2/3)
Molling, J.W. [45]	2007	Netherlands	HC = 33, HNSCC: <48 iNKT/10^6^ T Cells = 12, 48–242 iNKT/10^6^ T Cells = 23, >242 iNKT/10^6^ T Cells = 12	HC = 65 (44–83), HNSCC: <48 iNKT/10^6^ T Cells = 67 (52–83), 48–242 iNKT/10^6^ T Cells = 66 (45–85), >242 iNKT/10^6^ T Cells = 62 (45–83)	HC = 59.57%, HNSCC: <48 iNKT/10^6^ T Cells = 66.67%, 48–242 iNKT/10^6^ T Cells = 60.87%, >242 iNKT/10^6^ T Cells = 50%	CD3^+^Vα24^+^Vβ11^+^	the frequency of iNKT cells in peripheral blood, OS, RFS	5 (3/0/2)
Najera Chuc, A.E. [46]	2012	Mexico	AML = 28	-	-	CD3^+^Vα24^+^Vβ11^+^	OS	6 (3/0/3)
Shadman, M. [47]	2015	Iran	HC = 24, Gastric Cancer = 24	-	-	CD3^+^Vα24^+^Jα18^+^	the frequency of iNKT cells in peripheral blood	6 (3/1/2)
Sun, W. [48]	2015	USA	HC = 42, Lymphoma = 55	-	-	Vα24^+^Jα18^+^Vβ11^+^	the frequency of iNKT cells in peripheral blood	5 (2/0/3)
Tachibana, T. [49]	2005	Japan	Colorectal carcinoma, High NKT-cell infiltration = 65, Low NKT-cell infiltration = 38	-	High NKT-cell infiltration = 55.38%, Low NKT-cell infiltration = 76.32%	Vα24^+^Vβ11^+^	OS, RFS	6 (3/0/3)
Vela–Ojeda, J. [50]	2024	Mexico	HC = 60 Diffuse large B-cell lymphoma = 59	Diffuse large B-cell lymphoma = 60 (23–86)	Diffuse large B-cell lymphoma = 56%	CD3^+^Vα24^+^Vβ11^+^	the frequency of iNKT cells in peripheral blood	8 (3/2/3)
Xiao, Y.S. [51]	2013	China	HCC = 224	-	-	Vα24^+^	OS, RFS	6 (3/0/3)
Xu, Y.G. [52]	2010	China	HC = 34, Minimal Residual Leukemia = 36	HC = 51 (17–60), Minimal Residual Leukemia = 49 (22–75)	HC = 55.88%, Minimal Residual Leukemia = 44.44%	Vα24^+^	the frequency of iNKT cells in peripheral blood	7 (4/0/3)
Yanagisawa, K. [53]	2002	Japan	HC = 22, Solid tumor = 21	HC = 47.68 (24–85), Solid tumor = 61.71 (47–80)	HC = 54.55%, Solid tumor = 57.14%	Vα24^+^	the frequency of iNKT cells in peripheral blood	5 (2/0/3)
Yoneda, K. [54]	2005	Japan	HC = 30, Hematological tumor = 70	-	-	Vα24^+^	the frequency of iNKT cells in peripheral blood	5 (2/0/3)
Singh, A.K. [55]	2013	India	HC = 28 Oral squamous cell carcinoma = 38	HC = 47.73 ± 1.33, Oral squamous cell carcinoma = 51.8 ± 1.64	HC = 75% Oral squamous cell carcinoma = 76.3%	6B11^+^CD3^+^	the frequency of iNKT cells in peripheral blood	8 (3/2/3)
Bojarska–Junak, A. [56]	2018	Poland	HC = 28 CLL = 60	HC = 57 (36–83) CLL = 67 (46–87)	HC = 57.14% CLL = 51.7%	CD3^+^Vα24^+^Jα18^+^	the frequency of iNKT cells in peripheral blood	7 (4/0/3)
Chan, A.C. [57]	2014	Australia	HC = 47 Multiple myeloma = 24	-	-	αGalCer- CD1d tetramer^+^CD3^+^	the frequency of iNKT cells in peripheral blood	6 (3/0/3)
Cheng, X. [58]	2023	China	HC = 41 HCC = 32	HC = 41 (18–69) HCC = 51.5 (22–69)	HC = 78.04% HCC = 81.25%	PBS57/hCD1d tetramer^+^CD3^+^	the frequency of iNKT cells in peripheral blood and tissues	5 (2/0/3)
Dockry, E. [59]	2018	Ireland	HC = 13 Lung cancer = 9	-	-	CD3^+^Vα24^+^Jα18^+^	the frequency of iNKT cells in peripheral blood	5 (2/0/3)
Dou, R. [60]	2018	China	HC = 20 T-cell lymphomas = 13	-	-	PBS57/hCD1d tetramer^+^CD3^+^	the frequency of iNKT cells in peripheral blood	5 (2/0/3)
Ghnewa, Y.G. [61]	2017	Ireland	HC = 12 CLL = 13	-	-	CD3^+^Vα24^+^Jα18^+^	the frequency of iNKT cells in peripheral blood	6 (2/1/3)
Horinaka, A. [62]	2016	Japan	HC = 18 HNSCC = 32	HC = 47.5 (35–63) HNSCC = 67.0 (34–83)	HC = 50% HNSCC = 81.25%	CD3^+^Vα24^+^	the frequency of iNKT cells in peripheral blood	5 (2/0/3)
Hus, I. [63]	2017	Poland	HC = 20 B-cell non-Hodgkin’s lymphoma = 41	HC = 57 (36–83) B-cell non-Hodgkin’s lymphoma = 65 (24–81)	HC = 60% B-cell non-Hodgkin’s lymphoma = 56.98%	CD3^+^Vα24^+^	the frequency of iNKT cells in peripheral blood	6 (2/1/3)
Imataki, O. [64]	2008	Japan	HC = 10 Malignant lymphoma = 6	HC = 28 (24–42) Malignant lymphoma = 48 (36–63)	HC = 60% Malignant lymphoma = 50%	Vα24^+^Vβ11^+^	the frequency of iNKT cells in peripheral blood	5 (2/0/3)
Iyoda, T. [65]	2018	Japan	HC = 9 Multiple myeloma = 29	-	-	CD3^+^Vα24^+^Vβ11^+^	the frequency of iNKT cells in peripheral blood	6 (2/1/3)
Jarvis, E.M. [66]	2021	New Zealand	HC = 20 Prostate cancer = 36	HC = 71 Prostate cancer = 70.5	HC = 100% Prostate cancer = 100%	Vα24^+^Jα18^+^	the frequency of iNKT cells in peripheral blood	8 (3/2/3)
Jiang, F. [67]	2018	China	HC = 23 Multiple myeloma = 37	HC = 58 (44–74) Multiple myeloma = 62 (45–77)	HC = 60.87% Multiple myeloma = 59.5%	Vα24^+^Vβ11^+^	the frequency of iNKT cells in peripheral blood	9 (4/2/3)
Kenna, T. [68]	2003	Ireland	HC = 7 Hepatic metastases of colonic origin = 10	-	-	CD3^+^Vα24^+^Vβ11^+^	the frequency of iNKT cells in tissues	5 (2/0/3)
Kobayashi, K. [69]	2010	Japan	HC = 14 HNSCC = 12	-	-	CD3^+^Vα24^+^Vβ11^+^	the frequency of iNKT cells in peripheral blood	8 (3/2/3)
Konishi, J. [70]	2004	Japan	HC = 20 Lung cancer = 55	HC = 56.3 ± 19.3 Lung cancer = 63.8 ± 11.3	HC = 60% Lung cancer = 65.45%	Vα24^+^Vβ11^+^	the frequency of iNKT cells in peripheral blood	7 (3/1/3)
Lattanzi, G. [71]	2023	Italy	Colorectal cancer = 118	Colorectal cancer = 70 ± 12.5	Colorectal cancer = 54.24%	PBS57/hCD1d tetramer^+^CD3^+^	the frequency of iNKT cells in tissues	8 (3/2/3)
Li, J. [72]	2014	USA	HC = 28 Hodgkin’s lymphomas = 17 Marginal zone lymphoma = 9 Diffuse large B-cell lymphoma = 29 Mantle cell lymphoma = 9 Follicular lymphoma = 18	-	-	Vα24^+^Vβ11^+^	the frequency of iNKT cells in peripheral blood	6 (3/0/3)
Liu, D. [73]	2013	USA	HC = 10 Medulloblastoma = 10	-	-	6B11^+^CD3^+^	the frequency of iNKT cells in peripheral blood	5(2/0/3)
Lo Nigro, C. [74]	2016	Italy	Colorectal cancer, iNKT above median level = 21 iNKT above blow level = 20	-	Colorectal cancer = 56%	CD3^+^Vα24^+^Vβ11^+^	OS	6 (3/0/3)
Melo, A.M. [75]	2020	Ireland	HC = 17 Esophageal adenocarcinoma = 80 Squamous cell carcinoma = 28 Gastric cancer = 31	Esophageal adenocarcinoma = 64.5 (43–96) Squamous cell carcinoma = 64.2 (46–80) Gastric cancer = 68.4 (49–87)	Esophageal adenocarcinoma = 91.3% Squamous cell carcinoma = 64.3% Gastric cancer = 74.2%	CD3^+^Vα24^+^Jα18^+^	the frequency of iNKT cells in peripheral blood	6 (3/0/3)
Metelitsa, L.S. [76]	2004	USA	HC = 36 Neuroblastoma = 8	-	-	Vα24^+^Jα18^+^Vβ11^+^	the frequency of iNKT cells in peripheral blood, OS	5(2/0/3)
Motohashi, S. [77]	2002	Japan	HC = 20 Lung cancer = 60	HC = 65.6 ± 10.7 Lung cancer = 67.6 ± 7.8	HC = 50% Lung cancer = 71.67%	CD3^+^Vα24^+^Vβ11^+^	the frequency of iNKT cells in peripheral blood	5(2/0/3)
Nur, H. [78]	2013	Belgium	HC = 62 Multiple Myeloma = 55	-	-	αGalCer- CD1d tetramer^+^TCRβ^+^	the frequency of iNKT cells in peripheral blood	6 (3/0/3)
Stavrou, V. [79]	2023	UK	HC = 19 AML = 37	-	-	Vα24^+^Vβ11^+^	the frequency of iNKT cells in peripheral blood	5(2/0/3)
Weinkove, R. [80]	2013	New Zealand	HC = 30 CLL = 40	-	-	αGalCer- CD1d tetramer^+^6B11^+^	the frequency of iNKT cells in peripheral blood	6 (2/1/3)
Winkler, I. [81]	2020	Poland	HC = 20 Ovarian cancer = 24	HC = 50 (37–78) Ovarian cancer = 55 (44–80)	HC = 0% Ovarian cancer = 0%	CD3^+^Vα24^+^	the frequency of iNKT cells in peripheral blood and tissues, OS	7 (2/2/3)
Altomare, E. [82]	2012	Italy	HC = 22 Malignant mesothelioma = 30	HC = 58.5 ± 1.9 Malignant mesothelioma = 62.5 ± 1.8	HC = 86.36% Malignant mesothelioma = 76.67%	CD3^+^Vα24^+^Vβ11^+^	the frequency of iNKT cells in peripheral blood	7 (2/2/3)
Hirahara, Y. [83]	2024	Japan	HC = 17 Cervical cancer = 13	-	-	Vα24^+^	the frequency of iNKT cells in peripheral blood	5(2/0/3)
Klatka, J. [84]	2017	Poland	HC = 15 Laryngeal carcinoma = 50	HC = 61 (43–82) Laryngeal carcinoma = 60 (45–77)	HC = 80% Laryngeal carcinoma = 80%	Vα24^+^Jα18^+^	the frequency of iNKT cells in peripheral blood	6 (3/0/3)
Hishiki, T. [20]	2018	Japan	Neuroblastoma, Vα24-Jα18-positive = 53 Vα24-Jα18 null = 36	-	-	Vα24^+^Jα18^+^	OS	6 (3/0/3)
Lee, I.C. [22]	2013	China	HCC = 63	HCC = 63.7 ± 11.7	HCC = 74.6%	Vα24^+^Jα18^+^	RFS	6 (3/0/3)

HC: health control. HCC: hepatocellular carcinoma. HNSCC: head and neck squamous cell carcinoma. AML: acute myelogenous leukemia. ALL: acute lymphoblastic leukemia. CLL: chronic lymphocytic leukemia. OS: overall survival. RFS: relapse-free survival.

**Table 2:** The basic characteristics of the clinical trials.

Author	Year	Country	Sample Size	Age (Years) (Mean ± Standard Deviation or Median and Range)	Male (%)	Duration of Expansion	Cytokines for Expansion	iNKT Markers	Total Dose	Frequency of Infusion	Follow-Up Time	Endpoint	MINORS Score
Exley, M.A. [10]	2017	USA	Melanoma = 9	Melanoma = 61 ± 11	Melanoma = 77.8%	6–8 weeks	CD3 mAb and IL-2	Vα24^+^Jα18^+^	iNKT: 4–217 × 10^6^ cells (Divide into 3 equal portions)	3 × 2-weekly infusions	Median 63 months (53–85 months)	DCR	12
Gao, Y. [11]	2021	China	HCC = 10	HCC = 51.5 (37–60)	HCC = 90%	3 weeks	α-GalCer and IL2	CD3^+^Vα24^+^Vβ11^+^	iNKT: 5.04–1000 × 10^7^ cells (Divided into 10%, 30%, 60% of total dose)	3 consecutive days	Every 3 months from 12 weeks after infusion	DCR	12
Giaccone, G. [12]	2002	The Netherlands	Solid Tumors = 24	Solid Tumors = 54 (31–69)	Solid Tumors = 58.33%	-	-	CD3^+^Vα24^+^Vβ11^+^	α-GalCer (50–4800 ug/m^2^)	Days 1, 8, and 15 of a 4-weekly cycle	Median 123 days (range, 83–216 days)	the frequency of iNKT cells in peripheral blood, ORR, DCR	10
Ishibashi, F. [13]	2020	Japan	Lung cancer = 21	Lung cancer = 61.71 ± 11.1	Lung cancer = 80. 95%	1–2 weeks	GM-CSF, IL-2	Vα24^+^Vβ11^+^	α-GalCer-pulsed APC (1 × 10^8^, 5 × 10^8^, and 1 × 10^9^ cells)	1-, 2-week	5 weeks	ORR, DCR	9
Ishikawa, A. [14]	2005	Japan	Lung cancer = 11	Lung cancer = 69.0 (63–78)	Lung cancer = 72.73%	1–2 weeks	GM-CSF, IL-2	Vα24^+^Vβ11^+^	α-GalCer-pulsed dendritic cells (5 × 10^7^, 2.5 × 10^8^, and 1 × 10^9^ cells/m^2^)	2-, 3-, 8-, 9-week	12 weeks	ORR, DCR	10
Kunii, N. [15]	2009	Japan	HNSCC = 8	HNSCC = 60 ± 14.38	HNSCC = 62.5%	α-GalCer-pulsed APC (7 days), Vα24 NKT cells (2 weeks)	α-GalCer-pulsed APC (GM-CSF and IL-2), Vα24 NKT cells (IL-2 and α-GalCer)	Vα24^+^Vβ11^+^	α-GalCer-pulsed APC (1 × 10^8^ cells) and Vα24 NKT cells (5 × 10^7^ cells)	α-GalCer-pulsed APC (7-, 13 day), Vα24 NKT cells (14-day)	5 weeks	ORR, DCR	11
Motohashi, S. [16]	2006	Japan	Lung cancer = 11	Lung cancer = 67.33 ± 9.2	Lung cancer = 66.67%	2–3 weeks	α-GalCer, IL2	Vα24^+^Vβ11^+^	iNKT: 1 × 10^7^ and 5 × 10^7^ cells/m^2^	2-, 3-week	7 weeks	ORR, DCR	11
Toyoda, T. [17]	2020	Japan	Lung cancer = 35	Lung cancer = 59.5 (38–75)	Lung cancer = 68.8%	1–2 weeks	GM-CSF and IL-2	Vα24^+^Vβ11^+^	-	α-GalCer-pulsed APC (7-, 14-, 49-, 56-day)	2 years	ORR, DCR	11
Uchida, T. [18]	2008	Japan	HNSCC = 9	HNSCC = 59.22 ± 6.85	HNSCC = 88.89%	7 days	GM-CSF and IL-2	Vα24^+^Vβ11^+^	α-GalCer-pulsed APC: 1 × 10^8^ cells	1-, 2-week	9 weeks	ORR, DCR	12

HCC: hepatocellular carcinoma. HNSCC: head and neck squamous cell carcinoma. APC: antigen-presenting cells. ORR: objective response rate. DCR: disease control rate.

## Data Availability

The raw data supporting the conclusions of this article will be made available by the authors on request.

## Supplementary Materials

The following supporting information can be downloaded at https://www.mdpi.com/article/10.3390/curroncol33100579/s1, Table S1: PRISMA 2020 Checklist [85]; Figure S1: The forest plot showing the frequency of iNKT cells in circulating T cells between healthy controls and cancer patients (HKSJ adjust) [9,38,39,44,47,55,56,57,58,61,63,66,67,75,76,80,81,83,84]; Figure S2: The forest plot showing the frequency of iNKT cells in circulating T cells between healthy controls and cancer patients (subgroup analysis based on cancer type) [9,38,39,44,47,55,56,57,58,61,63,66,67,75,76,80,81,83,84]; Figure S3: The forest plot showing the frequency of iNKT cells in circulating mononuclear cells between healthy controls and cancer patients (HKSJ adjust) [35,37,40,42,43,48,52,53,60,62,64,65,70,72,73,79]; Figure S4: The forest plot showing the frequency of iNKT cells in circulating mononuclear cells between healthy controls and cancer patients (subgroup analysis based on cancer type) [35,37,40,42,43,48,52,53,60,62,64,65,70,72,73,79]; Figure S5: The forest plot showing the frequency of iNKT cells in circulating lymphocytes between healthy controls and cancer patients (HKSJ adjust) [36,41,54,57,59,77,82]; Figure S6: The forest plot showing the frequency of iNKT cells in circulating lymphocytes between healthy controls and cancer patients (subgroup analysis based on cancer type) [36,41,54,57,59,77,82]; Figure S7: The forest plot showing the number of circulating iNKT cells between healthy controls and cancer patients (HKSJ adjust) [12,39,43,45,50,52,54,59,61,69,77,78]; Figure S8: The forest plot showing the number of circulating iNKT cells between healthy controls and cancer patients (subgroup analysis based on cancer type) [12,39,43,45,50,52,54,59,61,69,77,78]; Figure S9: The forest plot showing the frequency of iNKT cells to T cells in non-tumor and tumor tissues (subgroup analysis based on cancer type) [38,58,68,71,81]; Figure S10: The forest plot showing higher frequency of iNKT cells and overall survival (subgroup analysis based on cancer type) [9,20,45,46,49,51,74,76,81]; Figure S11: The forest plot showing higher frequency of iNKT cells and relapse-free survival (HKSJ adjust) [22,45,49,51]; Figure S12: The forest plot showing higher frequency of iNKT cells and relapse-free survival (subgroup analysis based on cancer type) [22,45,49,51]; Figure S13: The forest plot showing the objective response rate of iNKT cell-related therapies (based on cancer type) [12,13,14,15,16,17,18]; Figure S14: The forest plot showing the objective response rate of iNKT cell-related therapies (based on iNKT cell therapy) [12,13,14,15,16,17,18]; Figure S15: The forest plot showing the disease control rate of iNKT cell-related therapies (based on cancer type) [10,11,12,13,14,15,16,17,18]; Figure S16: The forest plot showing the disease control rate of iNKT cell-related therapies (based on iNKT cell therapy) [10,11,12,13,14,15,16,17,18]; Figure S17: Publication bias in the frequency of iNKT cells in circulating T cells [9,38,39,44,47,55,56,57,58,61,63,66,67,75,76,80,81,83,84]. (A). Egger test (*p* = 0.024). (B). Funnel plot. (C). Trim-and-fill method; Figure S18: Publication bias in the frequency of iNKT cells in circulating mononuclear cells [35,37,40,42,43,48,52,53,60,62,64,65,70,72,73,79]. (A). Egger test (*p* = 0.025). (B). Funnel plot. (C). Trim-and-fill method; Figure S19: Publication bias in the frequency of iNKT cells in circulating lymphocytes [36,41,54,57,59,77,82]. (A). Egger test (*p* = 0.366). (B). Funnel plot; Figure S20: Publication bias in the number of circulating iNKT cells [12,39,43,45,50,52,54,59,61,69,77,78]. (A). Egger test (*p* = 0.278). (B). Funnel plot; Figure S21: Publication bias in the frequency of iNKT cells to T cells in tissues [38,58,68,71,81]. (A). Egger test (*p* = 0.557). (B). Funnel plot; Figure S22: Publication bias in overall survival [9,20,45,46,49,51,74,76,81]. (A). Egger test (*p* = 0.394). (B). Funnel plot; Figure S23: Publication bias in relapse-free survival [22,45,49,51]. (A). Egger test (*p* = 0.255). (B). Funnel plot; Figure S24: Publication bias in objective response rate [12,13,14,15,16,17,18]. (A). Egger test (*p* = 0.327). (B). Funnel plot; Figure S25: Publication bias in disease control rate [10,11,12,13,14,15,16,17,18]. (A). Egger test (*p* = 0.062). (B). Funnel plot; Figure S26: The leave-one-out method for the frequency of iNKT cells in circulating T cells [9,38,39,44,47,55,56,57,58,61,63,66,67,75,76,80,81,83,84]; Figure S27: The leave-one-out method for the frequency of iNKT cells in circulating mononuclear cells [35,37,40,42,43,48,52,53,60,62,64,65,70,72,73,79]; Figure S28: The leave-one-out method for the frequency of iNKT cells in circulating lymphocytes [36,41,54,57,59,77,82]; Figure S29: The leave-one-out method for the number of circulating iNKT cells [12,39,43,45,50,52,54,59,61,69,77,78].
